# A Review of Cyclic Phosphatidic Acid and Other Potential Therapeutic Targets for Treating Osteoarthritis

**DOI:** 10.3390/biomedicines11102790

**Published:** 2023-10-14

**Authors:** Tamotsu Tsukahara, Shigeyuki Imamura, Toshiro Morohoshi

**Affiliations:** 1 Department of Pharmacology and Therapeutic Innovation, Nagasaki University Graduate School of Biomedical Sciences, Nagasaki 852-8521, Japan; 2 SANSHO, Co., Ltd., Tokyo 103-0027, Japan; imamura@triton.ocn.ne.jp (S.I.); tmorohoshi@c-pa.jp (T.M.)

**Keywords:** cyclic phosphatidic acid, inflammation, osteoarthritis, lysophospholipid

## Abstract

Osteoarthritis (OA), a chronic degenerative joint disease, is the most common form of arthritis. OA occurs when the protective cartilage that cushions the ends of bones gradually breaks down. This leads to the rubbing of bones against each other, resulting in pain and stiffness. Cyclic phosphatidic acid (cPA) shows promise as a treatment for OA. In this article, we review the most recent findings regarding the biological functions of cPA signaling in mammalian systems, specifically in relation to OA. cPA is a naturally occurring phospholipid mediator with unique cyclic phosphate rings at the sn-2 and sn-3 positions in the glycerol backbone. cPA promotes various responses, including cell proliferation, migration, and survival. cPA possesses physiological activities that are distinct from those elicited by lysophosphatidic acid; however, its biochemical origin has rarely been studied. Although there is currently no cure for OA, advances in medical research may lead to new therapies or strategies in the future, and cPA has potential therapeutic applications.

## 1. Overview

According to the World Health Organization (WHO), osteoarthritis (OA) is one of the most common musculoskeletal disorders, affecting millions of people worldwide [1]. 

OA is a comprehensive joint disease that affects various joint tissues, manifesting as subchondral bone remodeling [2], meniscal degeneration [3], inflammation [4], fibrosis of the infrapatellar fat pad [5], and synovial membrane inflammation [6]. OA is a chronic joint condition characterized by the degeneration of the joint cartilage and underlying bone, leading to symptoms such as pain, stiffness, and reduced mobility. OA is more prevalent in older age groups than in younger age groups, and it is estimated that a significant percentage of people aged 60 years and older have OA to some degree [7]. While OA can affect both men and women, it may be more common in women, especially in certain joints such as the hands and knees [8]. The WHO emphasizes the importance of early diagnosis and appropriate management of OA. This includes a combination of non-pharmacological interventions (e.g., exercise, weight management) and pharmacological treatments (e.g., pain relievers, anti-inflammatory drugs) to control symptoms and improve quality of life for individuals with OA. Various drugs and medications are used to manage the symptoms and improve the quality of life of patients with OA. In some cases, biological drugs targeting specific inflammatory pathways may be prescribed, particularly for individuals with inflammatory OA or other underlying inflammatory conditions. Researchers have explored various regenerative therapies such as stem cell therapy and platelet-rich plasma (PRP) injections to promote repair and regeneration of damaged joint tissues [9,10]. These therapies offer new options for the treatment of OA. However, disease-modifying osteoarthritis drugs (DMOADs) such as hyaluronic acid or PRP injections can be costly, and managing medical bills associated with OA is a significant concern for individuals affected by this condition. The frequency and type of injection used can affect the overall cost. 

Ongoing research on the mechanisms underlying OA and development of innovative therapies will continue to shape the future of OA management. This review focuses on the most recent findings regarding the biological functions of cyclic phosphatidic acid (cPA) signaling in mammalian systems in relation to OA.

## 2. Medications for OA

Currently, treatment standards for OA are primarily limited to pain management, steroids, other anti-inflammatory drugs, physical therapy, and eventual joint replacement [11,12,13]. The most common symptoms of OA include joint pain, stiffness, and a decreased range of motion. These symptoms tend to worsen over time, particularly after periods of inactivity or excessive use of the affected joint. Several risk factors are associated with OA, including aging, genetics, joint injury or trauma, obesity, and certain joint abnormalities [14,15,16]. Repetitive stress on a joint, such as from certain occupations or sports activities, can also increase one’s risk of OA. Although there is no cure for OA, several treatment options are available to manage its symptoms and improve joint function. Corticosteroid and hyaluronic acid injections are two different types of treatments commonly used for various medical conditions, particularly in the fields of orthopedics and rheumatology [17]. Corticosteroids are potent anti-inflammatory drugs. Injection of such drugs into a joint or soft tissue can help reduce inflammation and alleviate pain. Hyaluronic acid is a natural substance found in joint fluids and cartilage. Viscosupplementation involves the injection of hyaluronic acid into the joint to improve lubrication and reduce pain [18]. However, recent research has found that viscosupplementation is not effective in significantly reducing pain or improving function [19]. Biological therapies are newer medications used for severe OA and include drugs such as DMOADs, PRP, and stem cell injections. DMOADs are a class of medications that aim to modify the underlying processes and progression of OA, rather than simply managing the symptoms; several potential DMOADs have been investigated in clinical trials and research studies. Potential DMOADs being explored include sprifermin, a growth factor that stimulates cartilage growth, and various agents targeting specific inflammatory pathways involved in OA [20,21,22]. PRP is a component of the blood that contains a high concentration of platelets. Platelets are rich in growth factors and bioactive proteins that play crucial roles in tissue repair and regeneration [23,24,25]. Additionally, injected stem cells are believed to have several potential mechanisms of action, including anti-inflammatory effects, cartilage repair, and pain reduction [26]. These treatments promote tissue repair and reduce inflammation. Although some patients report pain relief after the procedure, others are not helped by the injections [27]. The choice between corticosteroids and hyaluronic acid injections is made depending on the specific condition being treated, the patient’s medical history, and the physician’s recommendation [28]. Both corticosteroids and hyaluronic acid injections have their own unique mechanisms of action and are used for different purposes. Corticosteroids are powerful anti-inflammatory medications that work by suppressing the immune response and reducing inflammation in the body. They are commonly used for conditions involving acute or chronic inflammation. Hyaluronic acid is a natural component found in joint fluid and cartilage, which helps lubricate and cushion the joints. Hyaluronic acid injections are primarily used for conditions with characteristics of joint pain and reduced joint lubrication, particularly in OA [29]. It is important to note that the choice between corticosteroid and hyaluronic acid injections should be made by a qualified healthcare provider after a thorough evaluation of the patient’s medical history, the specific condition being treated, as well as a consideration of the potential benefits and risks associated with each treatment option. These drugs target various aspects of the disease, including cartilage protection, inflammation reduction, and joint function improvement, highlighting the existence of alternative and complementary therapies for OA that may have different profiles regarding treatment resistance, side effects, and costs, as well as acknowledging that not all patients respond equally to OA treatments. Some patients may experience limited or no improvement in symptoms despite trying various therapies. Side effects can vary from person to person and some individuals may experience minimal or no adverse effects. By addressing these factors in discussions about treatments for OA, healthcare providers can offer patients a more comprehensive understanding of their options and help them make informed decisions that align with individual circumstances and priorities. This approach promotes patient-centered care and better outcomes for individuals living with OA.

## 3. Inflammation and OA

**Figure 1:**
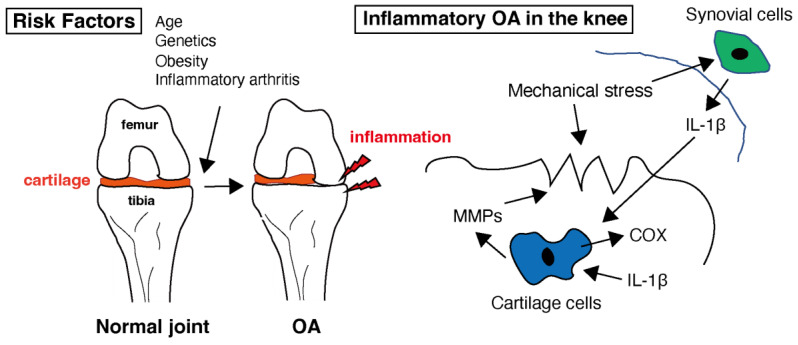
Healthy cartilage provides a smooth and lubricated surface within the joint, allowing for pain-free movement. Several risk factors that can increase the likelihood of developing osteoarthritis (OA) have been identified. While OA is primarily considered a non-inflammatory arthritis, low-level inflammation does play a role in the disease. In the context of OA, interleukin-1 beta (IL-1β) is of particular interest because it can contribute to the inflammation and tissue damage seen in affected joints. Inflammation in the joint can lead to the release of enzymes that further break down cartilage. Matrix metalloproteinases (MMPs) are involved in the breakdown of the extracellular matrix, which is the structural framework of tissues in the body, including cartilage in joints. Cyclooxygenase (COX) is an enzyme involved in the production of prostaglandins within the cartilage tissue.

OA is characterized by low-grade chronic inflammation of the affected joint [30]. Inflammation primarily involves synovium and joint tissues. Although not as severe or systemic as rheumatoid arthritis (RA), inflammation contributes to the symptoms and progression of OA. Inflammatory molecules, such as cytokines and chemokines, are produced in inflamed joint tissues [31]. As shown in Figure 1, these molecules promote cartilage degradation, inhibit cartilage repair, and contribute to joint pain and stiffness. Levels of several pro-inflammatory cytokines, e.g., interleukin-1 (IL-1), interleukin-6 (IL-6), and tumor necrosis factor-alpha (TNF-α), are increased in the synovial fluid and joint tissues of individuals with OA [32,33,34]. In healthy joints, there is a balance between proinflammatory and anti-inflammatory cytokines. In OA, this balance is disrupted by an increase in proinflammatory cytokines and a decrease in anti-inflammatory cytokines. This imbalance contributes to the ongoing inflammation and tissue destruction [35]. Cytokines, especially IL-1 and TNF-α, can sensitize pain receptors in the joint tissues, leading to increased pain perception in individuals with OA [36]. This heightened pain sensation can further reduce joint function and quality of life. These mediators in OA can also stimulate chondrocytes to produce enzymes, such as matrix metalloproteinases (MMPs) and aggrecanases [37]. MMPs are a family of enzymes that play crucial roles in tissue remodeling, particularly in the breakdown of the extracellular matrix (ECM). These enzymes, particularly MMP-1, MMP-3, and MMP-13, break down important ECM components such as collagen and proteoglycans, which are essential for cartilage integrity [38,39]. This degradation leads to the thinning and erosion of the cartilage in OA-affected joints. MMPs are produced by various cell types, including chondrocytes, synovial cells, and immune cells [40]. Inflammation in OA joints can stimulate the release of MMPs, further accelerating cartilage breakdown and contributing to joint damage [41]. MMPs are essential for normal tissue remodeling and repair. However, in OA, there is often an imbalance between MMP production and tissue repair mechanisms, leading to excessive matrix degradation and inadequate tissue regeneration [42]. The body contains natural MMP inhibitors, such as tissue inhibitors of metalloproteinases (TIMPs). In individuals with OA, the balance between MMPs and TIMPs may be disrupted, favoring MMP activity and cartilage breakdown [43]. This imbalance may contribute to the disease progression. Targeting MMPs is a potential therapeutic strategy for OA [44]. Developing drugs or interventions that can inhibit the activity of specific MMPs may help slow cartilage degradation and mitigate OA symptoms [45]. However, identifying effective and safe MMP inhibitors for clinical use remains a challenge. While MMPs are involved in the degradation of cartilage in OA and have been explored as therapeutic targets, developing effective MMP inhibitors has proven to be a complex task [45]. MMPs play various roles in tissue remodeling and homeostasis. Developing inhibitors that selectively target the MMPs involved in OA without affecting beneficial MMPs can be challenging. Many MMP inhibitors developed in the past have shown off-target effects, impacting other biological processes. The lack of specificity can lead to unwanted side effects and safety concerns [46]. MMP inhibitors may need to be used in combination with other OA treatments, such as non-steroidal anti-inflammatory drugs (NSAIDs) or disease-modifying OA drugs (DMOADs), to achieve optimal results. The interactions and safety of combination therapies need careful evaluation [47]. In some cases of OA, the synovial membrane becomes inflamed, leading to increased production of synovial fluid and joint swelling [6]. Synovial inflammation can exacerbate joint pain and discomfort. Additionally, inflammatory processes within the joint can lead to pain, which is a hallmark of OA. Inflammation contributes to pain perception in patients with OA, and it accelerates the progression of OA by promoting cartilage damage and joint deterioration [48]. This leads to a vicious cycle of inflammation and joint damage. Cytokines are important mediators of inflammation and tissue damage in patients with OA. Thus, managing inflammation and restoring the balance between pro- and anti-inflammatory cytokines are important aspects of the treatment and management of this condition.

## 4. Chondroitin Sulfate Synthase and Osteoarthritis

Chondroitin sulfate (CS) is a naturally occurring glycosaminoglycan (GAG) found in cartilage and connective tissues of the human body that plays a critical role in maintaining the structural integrity and lubrication of joints [49,50,51,52]. CS is one of the substances that makes up the ECM of the cartilage, providing it with cushioning and shock-absorbing properties. Researchers have explored the role of CS in OA, primarily focusing on its potential therapeutic implications [53]. CS is available as a dietary supplement and is often used by individuals with OA to manage their symptoms. This supplement is believed to provide the building blocks necessary for cartilage repair and maintenance. CS plays a role in joint health by supporting the proper function of synovial fluid, which lubricates and nourishes the joints [54]. It is also believed to have anti-inflammatory and cartilage-protective properties [55]. This may help reduce cartilage degradation and inhibit enzymes that break down the ECM [56]. In some cases, CS supplements, such as glucosamine, are used in combination with other treatments for OA. These combination therapies address multiple aspects of OA, including pain relief, inflammation reduction, and cartilage support. Clinical studies examining the effectiveness of CS supplementation in OA have yielded mixed results. Some studies have suggested that CS provides mild to moderate pain relief and improvements in joint function, whereas others have not shown significant benefits [49]. The effectiveness of CS may depend on factors such as OA severity and individual responses. However, this area of research is still in its early stages, and more studies are needed to understand the feasibility and effectiveness of such interventions. CS synthase is an enzyme involved in the biosynthesis of CS. Researchers have investigated whether modulating the activity of CS synthase or the production of CS could be a potential therapeutic approach for OA. Chondroitin sulfate is a major component of cartilage and plays a crucial role in maintaining its structural integrity and function. Modulating the activity of CS synthase, the enzyme responsible for synthesizing chondroitin sulfate, or the overall production of CS could impact the quality of cartilage and potentially slow down the progression of OA [57]. Chondroitin sulfate synthase 1 (CHSY1), chondroitin sulfate synthase 2 (CHSY2), and chondroitin sulfate synthase 3 (CHSY3) are different isoforms of the CS synthase enzyme family, each with its own unique role in the biosynthesis of CS. CHSY1 is one of the isoforms responsible for the sulfation of CS. It catalyzes the transfer of sulfate groups to specific positions in the core protein structure during CS biosynthesis. CHSY1 is involved in the early stages of CS chain formation [58]. CHSY2 plays a role in the sulfation process and contributes to the addition of sulfate groups to CS chains. CHSY2 may have distinct substrate preferences or enzymatic activities compared with CHSY1 [59]. CHSY3 is the third isoform in this enzyme family. Similar to CHSY1 and CHSY2, it participates in the sulfation of CS chains by adding sulfate groups to specific positions in the core protein structure [60,61]. CHSY3 may have unique characteristics and functions in the CS biosynthesis pathway [59]. These isoforms are collectively responsible for the sulfation pattern of CS molecules, which varies depending on the tissue and specific functions within the body [59]. Understanding the functions of CHSY1, CHSY2, and CHSY3 is crucial for unraveling the complexity of CS biosynthesis and its implications in tissue health and various diseases, including those related to connective tissues and joints. Furthermore, CS synthase or chondroitin polymerizing factor 2 (CHPF2) is an enzyme involved in the biosynthesis of CS [62]. CHPF2 plays a crucial role in the elongation and polymerization of CS chains, which are important for the structural integrity and function of these tissues [62]. CS N-Acetylgalactosaminyltransferase (CSGALNACT) is another enzyme involved in the biosynthesis of CS. CSGALNACT catalyzes the transfer of N-acetylgalactosamine to the core protein structure during CS biosynthesis [63]. Researchers have investigated whether modulating the activity of CS synthase or the production of CS could be a potential therapeutic approach for OA [57]. In addition to the potential therapeutic use of CS for OA, it is crucial to address side effects and interactions, as well as provide practical guidance for patients. CS is generally considered safe, but it is essential for patients to consult with their healthcare provider before starting to use any new supplement, especially if they have allergies or are taking other medications [64]. OA is a chronic condition, and long-term management is essential. Patients should be aware that supplements like CS may need to be taken consistently for sustained benefits. Comparing the effectiveness and potential advantages or disadvantages of CS supplementation with other treatments for OA, such as glucosamine or NSAIDs, provides valuable information for patients and healthcare providers when considering treatment options [65]. Glucosamine is a natural compound found in the body, specifically in the fluid surrounding the joints. It is commonly used as a dietary supplement for the management of OA, a common degenerative joint condition [66]. Glucosamine supplements are often marketed as a way to support joint health and alleviate the symptoms of OA. These supplements are available in various forms, including glucosamine sulfate, glucosamine hydrochloride, and N-acetylglucosamine [67]. Consultation with a healthcare provider is essential to determine the most appropriate treatment plan for each patient’s specific needs and circumstances. Additionally, continuous monitoring and adjustments to the treatment plan may be necessary to achieve the best outcomes for OA management.

## 5. Overview of Cyclic Phosphatidic Acid 

Lipids are versatile molecules with different chemical structures and properties, and researchers have harnessed the properties of lipids to create drugs and drug delivery systems for different therapeutic purposes. Cyclic phosphatidic acid (cPA) is a unique phospholipid with a cyclic structure. This cyclic ring differentiates it from typical linear phosphatidic acids including lysophosphatidylcholine (LPC) and lysophosphatidic acid (LPA) [68,69]. The first cPA family member was originally isolated from slime mold and designated as physarum lysophosphatidic acid [70]. Several cPA activities have been attributed to albumin-associated lipid factors. Autotaxin (ATX) was initially identified as an enzyme secreted by cancer cells and found to stimulate cell motility, i.e., “taxis” [71]. cPA generation by autotaxin (ATX) under nonphysiological conditions was first reported in 2006 [72]. ATX is a lysophospholipase D and converts LPC into LPA [73,74,75]. LPA is a bioactive lipid that plays a role in inflammation [76]. ATX and the LPA it generates are involved in several physiological and pathological processes, including inflammation, angiogenesis, fibrosis, and cancer progression [77,78,79,80]. LPA is a potent chemoattractant that can attract immune cells, such as neutrophils and monocytes, to sites of inflammation [81]. This is important for the immune response against infections or tissue damage. Thus, ATX in LPA production can contribute to the recruitment of immune cells to inflamed tissues [82]. LPA can also stimulate the production of pro-inflammatory cytokines, such as IL-6 and TNF-α, by immune cells. These cytokines further amplify the inflammatory response. Excessive LPA signaling can lead to fibrosis, which is the formation of excess fibrous connective tissue [83]. Fibrosis is characterized by excessive accumulation of ECM proteins, e.g., collagen, in tissues, leading to tissue scarring and dysfunction [84,85]. This can occur in various organs and impair their function. LPA can induce fibrosis-associated inflammation [86]. Inflammatory cells, such as macrophages, can release LPA, and LPA, in turn, can recruit and activate immune cells, perpetuating the inflammatory response and contributing to fibrosis [87]. The signaling pathways triggered by LPA are complex and can have both pro-inflammatory and pro-survival effects [88]. Because of their involvement in various diseases, particularly cancer and inflammatory conditions, ATX and LPA have been investigated as potential therapeutic targets for OA. By inhibiting ATX, these inhibitors decrease LPA levels in the body, which can have therapeutic effects in certain medical conditions. ATX inhibitors have been explored as potential treatments for conditions such as fibrosis, cancer, and autoimmune diseases involving dysregulated LPA signaling [89]. The development of ATX inhibitors is an active area of research. Several compounds with inhibitory activity against ATX have been investigated in preclinical and clinical studies [90]. These inhibitors can be small molecules or biologics designed to disrupt the enzymatic activity of ATX or its interactions with its substrates [91]. cPA is also a physiological constituent of human serum [92]. Its stability allows cPA to function as a bioactive lipid mediator in various physiological processes [93]. cPA shows several unique actions compared with those of LPA. cPA inhibits cell proliferation, whereas LPA stimulates cell proliferation, migration, and differentiation [94]. cPA suppresses cancer cell invasion and metastasis by inhibiting ATX and transient activation of low-molecular-weight GTPases and RhoA [95]. Additionally, cPA can modulate ATX activity, affecting LPA levels [96] and, consequently, inflammation [97,98]. However, it is important to note that the metabolic stability of cPA can vary depending on factors such as the specific tissue or cell type, presence of enzymes or other molecules that may degrade it, and the local microenvironment. cPA has been implicated in various cellular processes and has important roles in cell signaling, cell growth, and differentiation [99,100,101,102,103,104]. It acts as a potent signaling molecule in various physiological contexts. The synthesis of cPA is tightly regulated, and its levels can be influenced by various cellular signals and stimuli. For example, certain growth factors, hormones, and neurotransmitters can modulate the activity of glycerophosphodiesterase 7 (GDE7) and thus affect cPA levels in the cell [105]. GDE7 suppresses the peroxisome proliferator-activated receptor gamma (PPARγ) pathway, suggesting that cPA functions as an intracellular lipid mediator. PPARγ is a type of nuclear receptor protein that plays a crucial role in regulating gene expression and is primarily involved in the control of lipid metabolism and glucose homeostasis [106]. Our previous studies have demonstrated that cPA negatively regulates PPARγ function by stabilizing the binding of the co-repressor protein, a silencing mediator of retinoic acid, and the thyroid hormone receptor [103]. We also showed that cPA prevents neointima formation, adipocyte differentiation, lipid accumulation, and upregulation of PPARγ target gene transcription [103].

Moreover, 2carba-cyclic phosphatidic acid (2carba-cPA) is a modified form of cPA [107], in which the phosphate group in cPA is replaced with a carba linkage, a carbon-carbon bond. This modification eliminates the negative charge typically associated with the phosphate group in cPA and can considerably affect the properties of a molecule and its biological activities [96,108,109,110,111]. One of the key features of 2carba-cPA is its enhanced stability compared to natural cPA. This stability allows it to persist longer in biological systems, making it a valuable tool for research and potential therapeutic applications. Like natural cPA, 2carba-cPA can interact with specific receptors, including G protein-coupled receptors (GPCRs), and initiate intracellular signaling cascades [112]. It may modulate various cellular processes, including cell proliferation, migration, and calcium signaling, depending on the cell type and receptor subtype involved. Research suggests that 2carba-cPA may have anti-cancer properties [112]. Preclinical studies have shown that 2carba-cPA inhibits the growth and metastasis of cancer cells. The stability and ability of 2carba-cPA to interfere with cancer cell signaling pathways make it a potential candidate for cancer therapy research. Some studies indicate that 2carba-cPA may have neuroprotective effects [109]. Therefore, its ability to protect neurons and potentially mitigate neurodegenerative diseases could be further explored. Some studies have shown the anti-inflammatory properties of 2carba-cPA [113], indicating its potential to modulate immune responses and inflammation-related diseases [114]. Research suggests that 2carba-cPA may play a role in metabolic regulation, including the control of lipid metabolism and glucose homeostasis, which could have implications for the treatment of metabolic disorders like diabetes and obesity. Due to its stability and promising biological activities, 2carba-cPA is a candidate for therapeutic development that may be used in drug discovery and development for conditions such as cancer, neurodegenerative diseases, inflammation-related disorders, and metabolic disorders.

## 6. In Vitro and In Vivo Studies of cPA as a Therapy for OA

**Figure 2:**
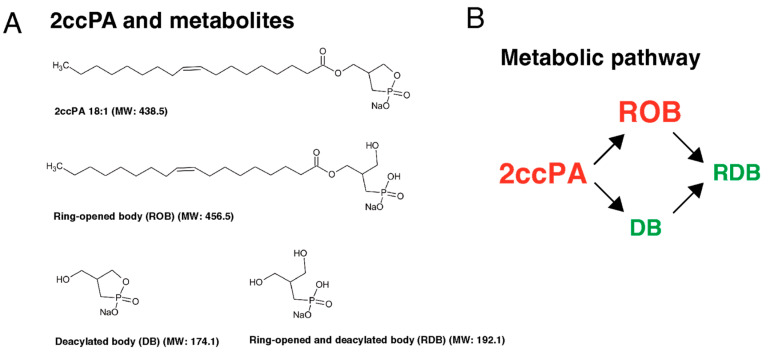
(**A**) Chemical structures of 2-carba-cyclic phosphatidic acid (2ccPA), ring-opened body (ROB), deacylated body (DB), and deacylated ring-opened body (RDB). Our previous ADME studies (Study No. AE-8545-G, Sekisui Medical Co., Ltd., Tokyo, Japan) described 2ccPA and its metabolites in lavage fluid of knee joints after a single intra-articular administration of [^14^C]- 2ccPA (dose: 0.5 mg/kg) in non-fasted male rabbits. Thirty minutes after administration, 2ccPA accounted for 84.4% of the knee joint lavage fluid, whereas DB and ROB (green text), detected as metabolites, accounted for 6.1% and 5.3%, respectively. (**B**) Proposed metabolic pathways of 2ccPA in a rabbit model. 2ccPA and ROB (red text) exhibited anti-inflammatory and chondroprotective activities.

**Figure 3:**
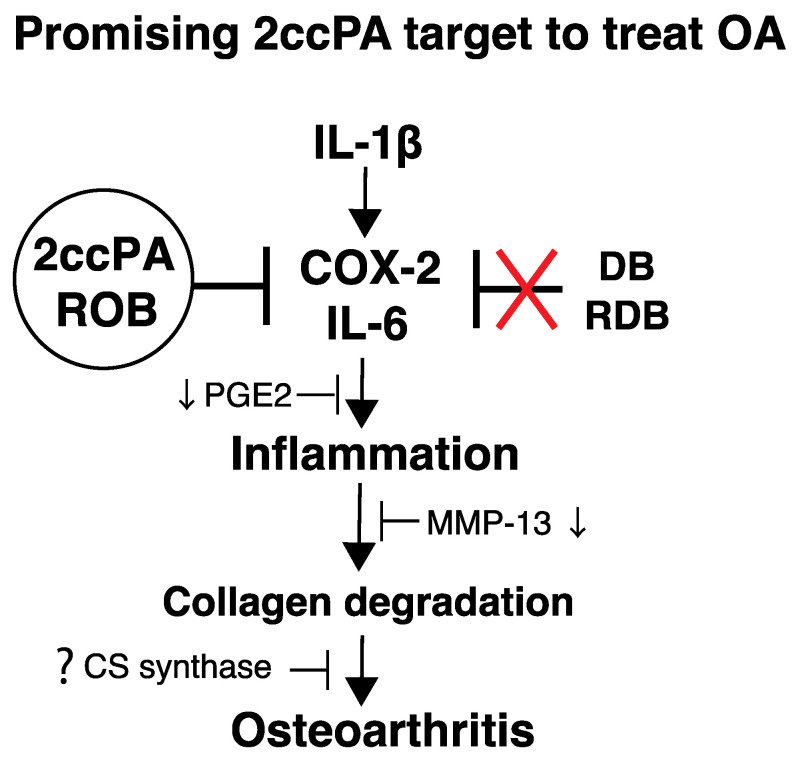
2ccPA and ring-opened body (ROB) affect PGE2 and interleukin-6 (IL-6) levels by inhibiting the mRNA and protein expression of Cyclooxygenase-2 (COX-2) and IL-6. However, it is still unknown how CS synthase affects 2ccPA dependent regulation. The intactness of 2ccPA and ROB is crucial for its anti-inflammatory effects on IL-1β-mediated inflammation. In addition, matrix metalloproteinases-13 (MMP-13) is an important collagenase and a critical OA target gene, and 2ccPA and ROB significantly suppress MMP-13 mRNA and protein expression. This study provides evidence that 2ccPA and ROB could be implemented as novel therapeutic agents for OA.

cPA has been detected in mammalian biological fluids, including human serum albumin (HSA). HSA is the most abundant plasma protein in human blood and is a major transporter for delivering lipids, including cPA [115]. However, extracellular cPA is hydrolyzed by phosphodiesterase and phospholipase, which leads to LPA formation [116], and the cleavage of fatty acids by phospholipase may limit the effective half-life of cPA. Among these novel compounds, carba derivatives of cPA, in which the phosphate oxygen is replaced with a methylene group at either the sn-2 or sn-3 position, shows a much more potent inhibitory effect than cPA [107]. One of the carba derivatives of cPA, 2ccPA, is a stabilized derivative compound of cPA in which the phosphate oxygen is replaced with a methylene group at the sn-2 position [69]. OA is the leading cause of functional loss and disability among older adults, with a substantial burden on both individuals and society [117]. Inflammatory cytokines, chemokines, and other inflammatory mediators are produced by the synovium and chondrocytes and can be detected in the synovial fluid of patients with OA [118]. OA results from the interaction of multiple mechanisms. Inflammation is a major factor associated with cartilage loss and other OA symptoms, including joint pain, swelling, stiffness, and synovitis [41]. Synovitis, which involves the infiltration of mononuclear cells into the synovial membrane and the production of pro-inflammatory mediators, including IL-1β, TNF-α, and chemokines, is common during early- and late-stage OA [4]. One of the important functions of IL-1β in the context of joint health and cartilage biology is its ability to stimulate chondrocytes. IL-1β stimulates chondrocytes to release proteolytic enzymes that drive cartilage destruction [119] and also contributes to the pain and symptoms associated with OA. It sensitizes the nerve endings in the joint, making the joint more sensitive to pain signals. This can result in increased pain and stiffness and decreased joint function. Recent studies suggest that the IL-1β inhibitor canakinumab shows promise as a treatment for OA [120,121]. IL-1β inhibitory effects give this monoclonal antibody its potential as an anti-osteoarthritis agent. Inflammation is a natural and essential component of the immune response; however, chronic or excessive inflammation can lead to various health problems, including OA. To investigate the effect of IL-1β and the possibility of treatment for OA, we applied 2-carba-cyclic phosphatidic acid (2ccPA) and its derivatives to human chondrocytes [122]. We found that cPA exhibits anti-inflammatory and chondroprotective activities [110,122]. Recently, a phase I human study was conducted to assess the tolerability and pharmacokinetics of carba derivatives of cPA. This novel compound of carba derivatives of cPA showed promising results (NCT05807529, ClinicalTrials.org) and is expected to enter phase II trials. This phase I/II study aims to evaluate the safety of single doses of 2ccPA (Phase I), as well as the safety and efficacy of multiple doses of 2ccPA (Phase II) in patients with OA of the knee. These reports suggest that 2ccPA and its ring-opened body (ROB) derivative (Figure 2) significantly suppressed IL-1β-induced upregulation of IL-6, MMP-13, and COX-2, as well as the degradation of type II collagen and aggrecan (Figure 3). However, the other two derivatives, namely the deacylated and ring-opened deacylated bodies, showed little effect on an IL-1β-exposed human chondrosarcoma cell line [122].

## 7. Current Treatment for OA

The treatment of OA is primarily focused on relieving pain, improving joint function, and minimizing disability. Current treatment options include NSAIDs, such as celecoxib, which selectively inhibits cyclooxygenase-2 (COX-2), and other pain relief medications that do not affect disease progression [123]. COX-2 is primarily associated with the production of prostaglandins (PG) during inflammatory responses. When tissues are injured or inflamed, COX-2 expression is upregulated, leading to increased PG production. PGs are signaling molecules that promote inflammation, pain, and fever. They also play a role in regulating the blood flow to injured tissues. Some medications specifically target COX-2 to reduce inflammation and pain while minimizing the potential gastrointestinal side effects associated with COX-1 inhibition [124]. These drugs are used to treat conditions such as OA, RA, and acute pain. However, a recent study suggested that COX-2 inhibitors are associated with an increased risk of adverse gastrointestinal and cardiovascular events, including hypertension, heart failure, and edema [125]. It is important for individuals to discuss the use of COX-2 inhibitors and other medications with their healthcare providers, particularly if they have underlying medical conditions or are taking other medications.

## 8. Conclusions and Future Perspectives

The future of OA treatment will likely involve several key developments and trends aimed at improving the prevention, diagnosis, treatment, and management of this common joint condition. OA is a multifaceted condition that affects individuals, communities, and healthcare systems. Understanding and addressing the different facets of OA are essential for effective prevention, management, and research of OA. The development of new drugs for OA is an ongoing area of research and innovation in medicine. Although no cure exists for OA, several approaches are being explored for drug development to manage symptoms, slow disease progression, and improve patients’ quality of life. Researchers have investigated the development of synthetic compounds or analogs that mimic the effects of cPA in OA treatment [110]. It is important to note that the development of drugs targeting specific lipid molecules such as cPA can be challenging, and the path to clinical use typically involves extensive preclinical research and clinical trials to establish safety and efficacy. Lipids are diverse organic molecules that play critical roles in cellular structure, energy storage, and signaling. Targeting specific lipids can have therapeutic benefits in a range of diseases. Lipid-based drug development continues to be an active area of research, with the potential for new therapies and drug delivery systems in OA.

## Data Availability

https://clinicaltrials.gov/study/NCT04229394 (accessed on 1 August 2023).
